# SIM2l attenuates resistance to hypoxia and tumor growth by transcriptional suppression of *HIF1A* in uterine cervical squamous cell carcinoma

**DOI:** 10.1038/s41598-017-15261-4

**Published:** 2017-11-06

**Authors:** Kanako Nakamura, Masayuki Komatsu, Fumiko Chiwaki, Takashi Takeda, Yusuke Kobayashi, Kouji Banno, Daisuke Aoki, Teruhiko Yoshida, Hiroki Sasaki

**Affiliations:** 1 Department of Obstetrics and Gynecology, Keio University School of Medicine, Tokyo, Japan; 2 Department of Translational Oncology, National Cancer Center Research Institute, Tokyo, Japan; 3 Fundamental Innovative Oncology Core Center, National Cancer Center Research Institute, Tokyo, Japan

## Abstract

Despite chemoradiotherapy being one of the most important modalities in advanced cervical cancer, there is a lack of both usable biomarkers to predict treatment outcome and of knowledge about the mechanism of refractoriness to the therapy. Here we identified a transcriptional factor Single-minded homolog 2 (SIM2) as an independent predictive biomarker for uterine cervical squamous cell carcinoma (CvSCC). The retrospective study showed that high expression level of SIM2 was correlated to good survival in CvSCC patients. SIM2 knockdown in CvSCC cell lines showed resistance to hypoxia with increased expression of *HIF1A* and its target genes. Loss of SIM2 also caused growth promotion, resistance to ROS, and radiation in 3D culture. Furthermore, SIM2 knockdown suppressed tumor growth with increased HIF-1α expression and angiogenesis *in vivo*. On the other hand, SIM2 long isoform (SIM2l)-overexpressed cells had contrary results, indicating the long isoform plays a key role for maintenance of these phenotypes. These data indicated that SIM2l has a potential to be precision medicine for CvSCC patients and that anti-angiogenesis therapy might be usable for SIM2l^Low^ poor survivors.

## Introduction

Among females, cervical cancer is one of the four most common malignancies, accounting for 528,000 new cases in 2012 and the fourth most common cause of cancer deaths worldwide, with an estimated 266,000 deaths in 2012 which are account for 7.5% of gynecological cancers^1^. A salient aspect of cervical cancer is chronic infection by various types of human papillomavirus (HPVs) which trigger carcinogenesis via integration of E6 and E7 oncogenes into the genome of host keratinocytes^2,3^. While cytology and vaccination-based cervical screening programs contribute to the amelioration of both incidence and mortality, the uptake of HPV vaccine has been poor, with 1–4% coverage of women worldwide and fewer in less-developed countries^4^. Indeed, death in less-developed region makes up more than 90% of the total^1^. Moreover, currently-used bivalent vaccines only target to HPV types 16 and 18, accounting for 70% of total cases, whereas 30% of patients who are infected by other types of HPVs result in incomplete protection from cervical cancer^5,6^. Prognoses of cases with early stages are reported to be relatively favorable (5-year survival rate of stage ΙA, ΙB, and ΙIA is 100%, 70–85%, and 70–85%, respectively), whereas those of advanced stages are still poor (5-year survival rate of stage ΙII and stage ΙV is 30–50% and 5–15%, respectively)^7^. Cervical squamous cell carcinomas (CvSCCs) account for approximately 75–80% of all cervical cancers and their primary management is well-established by each clinical stage. Surgery or radiotherapy alone is recommended for patients with early stages, and radiotherapy with/without cisplatin-based concurrent chemotherapy for patients with advanced stages^8,9^. However, the rates of distant and local relapse during 5 years after chemoradiotherapy are 14% and 19%, respectively^10^. Thus, a new modality to discover chemoradio-sensitive or -resistant biomarkers and understand their biological functions in effectiveness against chemoradiotherapy in cervical cancer is of great significance.

Single-minded homolog 2 (SIM2), a member of the basic HLH (helix-loop-helix)-PER-ARNT-SIM (bHLH-PAS) transcription factors, is identified within a Down’s syndrome-crucial region of chromosome 21^11–13^. Upon binding to aryl hydrocarbon receptor nuclear translocator (ARNT), which is a member of the same bHLH-PAS family and also forms a homodimer or heterodimer with aryl hydrocarbon receptor (AhR) or Hypoxia-inducible factor 1α (HIF-1α), SIM2 binds to central midline element (CME) in the regulatory region of target genes and then acts as a transcriptional repressor via C-terminal trans-repression domains^11,14^. During early fetal life, SIM2 expresses in the central nerve system and contributes to the formation of the brain structure necessary for learning and memory process, which can be a good explanation for symptoms of Down’s syndrome^15^. Aberrant expression of SIM2 has been reported in several types of cancers including prostate, colon, and pancreatic cancer^16–19^. Especially in prostate cancer, various studies have reported an increased expression of SIM2 and its contribution to tumor progression and aggressiveness^20,21^. The *SIM2* mRNA has two alternative splicing variants, SIM2 short (*SIM2s*) and SIM2 long (*SIM2l*). Both proteins share a nuclear-localization sequence, dimerization and repression domains, whereas SIM2l exclusively has 44 amino acids at the C terminus^11^. Current knowledge about misregulation of SIM2 in cancer is obtained from studies regarding SIM2s. Indeed, knockdown of SIM2s results in induction of cell-differentiation and apoptosis in colon cancer^18^. Likewise, SIM2s knockdown suppresses invasion of glioma cells through mesenchymal-epithelial transition^22^. In contrast to the oncogenic function mentioned above, SIM2s behaves as a tumor suppressor, which inhibits epithelial-mesenchymal transition and represses growth and invasion in breast cancer^23–25^. In addition to these facts that SIM2s functions differently by cancer types, the authentic function of SIM2l is still missing pieces of the puzzle. Since SIM2 has a Jekyll-and-Hyde character and role allotment of its splicing variants is still unclear, it is important to integrate their *bona fide* roles by cancer type.

In this study, we discovered SIM2 expression is an independent predictive biomarker for CvSCC patients who received chemoradiotherapy. A series of gene knockdown and overexpression experiments revealed that SIM2l acts as a tumor suppressor gene via transcriptional suppression of *HIF1A* and decreased radiation resistance and tumor growth in CvSCC.

## Results

### Decreased expression of SIM2 is a worse prognosis factor in cervical squamous cell carcinoma (CvSCC) patients

**Figure 1:**
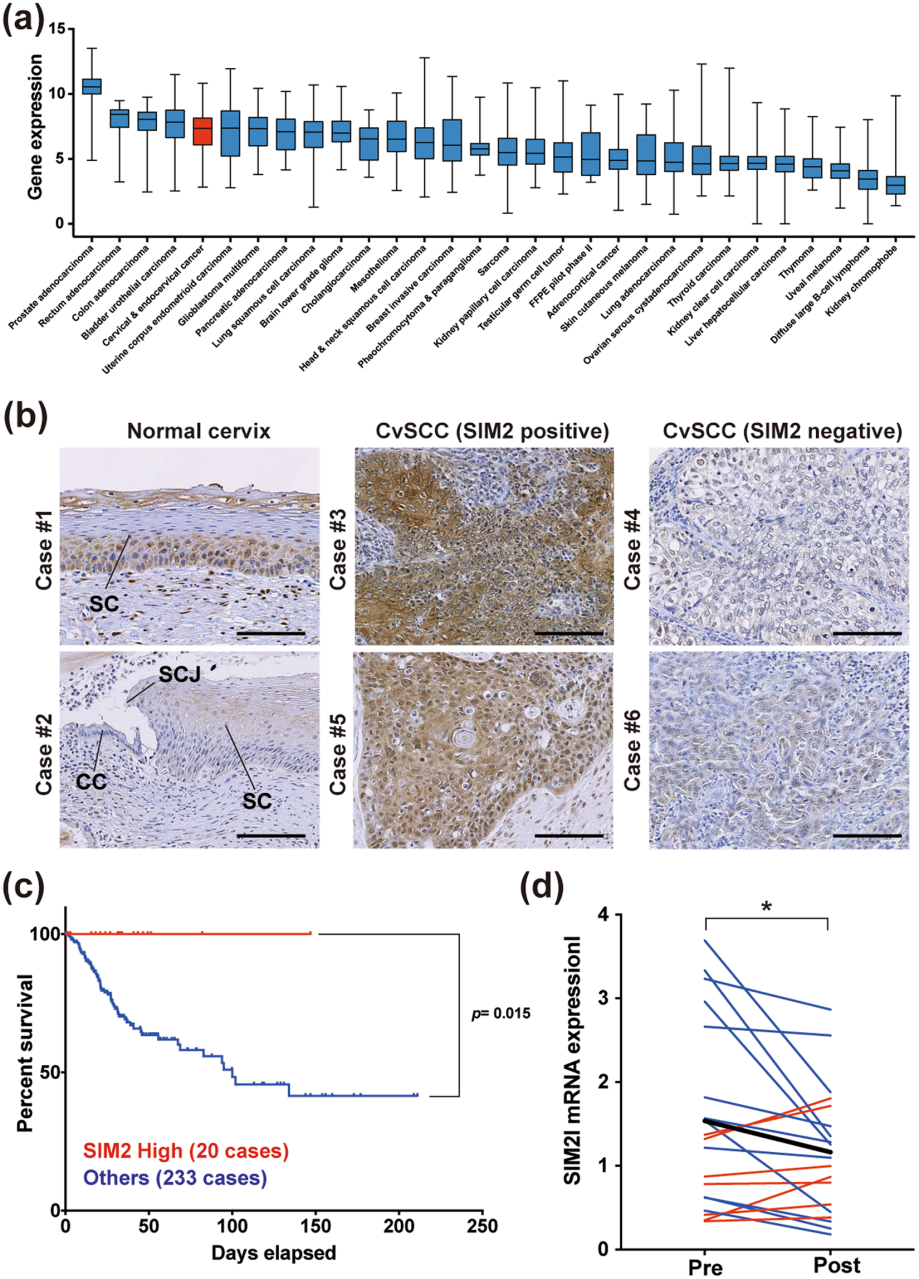
SIM2 is an independent predictive marker for cervical squamous cell carcinoma. **(a)** *SIM2* mRNA expression in pan cancers from TCGA data set. A box plot representing the median and ranges of normalized *SIM2* mRNA expression. **(b)** Representative immunohistochemistry of SIM2l in normal cervix and CvSCC tissue. SC, squamous cells; SCJ, squamocolumnar junction; CC, columnar cells. Each bar represents 100 μm. **(c)** Overall survival of CvSCC patients classified by *SIM2* mRNA expression. SIM2 High indicated the case which *SIM2* expression was more than double the average of 248 cases. Each dot represents censored patients. *P*-value was calculated by log-rank analysis. **Represents *p* < 0.01. **(d)** Expressional change of *SIM2l* mRNA encoding a long isoform by radiotherapy in each CvSCC patient. Red lines indicated increased expression after radiotherapy. Blue lines indicate decreased expression after radiotherapy. Bold line indicated an average expression of 18 patients. Data was analyzed by student t-test. *Represents *p* < 0.05.

SIM2 has been reported to be over-expressed in prostate cancer^20,21^, whereas its expression is decreased in esophageal squamous cell carcinoma compared to normal tissue^26^, indicating *SIM2* regulation is distinct by cancer types. About this notion, we first investigated the *SIM2* mRNA level in CvSCCs among various types of cancer by RNA sequencing dataset that consisted of 8449 cancer patients from The Cancer Genome Atlas (TCGA) database (Fig. 1a). *SIM2* mRNA expression was the highest in prostate cancer, and CvSCC was the fifth highest in all cancers. In CvSCC, *SIM2* mRNA levels varied by cases, indicating the existence of distinctive patients with low or high *SIM2* mRNA expression. We next analyzed SIM2l protein expression of CvSCC and normal cervix by immunohistochemistry (Fig. 1b). SIM2l was not expressed in the squamocolumnar junction (SCJ) epithelia that are the HPV-related and preneoplastic epithelia but expressed in parabasal cell layers of normal cervix. Although a major subset of CvSCCs expressed no/low SIM2l protein, we found a subset that expressed SIM2l protein aberrantly and highly. Consistent with these observations, *SIM2* mRNA expression was lower than normal cervix by expression analysis using Gene Expression Omnibus database (GEO) (Supplementary Figure S1a). We then evaluated the relationship between *SIM2* mRNA expression and clinical outcome using another TCGA dataset including RNA sequencing data of 248 CvSCC patients. In this data, 7.9% (20/253) cases were classified into a “SIM2 High” group whose *SIM2* expression is more than two-fold higher than the average (Supplementary Figure S1b). Kaplan-Meier analysis revealed that overall survival (OS) of cases with the “SIM2 High” in *SIM2* mRNA levels is significantly longer than others (Fig. 1c). In contrast, there was almost no aberrant expression of *ARNT*, which encodes a key dimerization partner of SIM2 necessary for its transcriptional repressor function (Supplementary Figure S1c). These data suggested that SIM2 plays an independent good prognosis factor in CvSCC patients. To reveal how SIM2 affects a prognosis in CvSCC patients, the relationship between *SIM2* mRNA levels and clinical features was also examined by the same TCGA dataset (Table 1). *SIM2* mRNA levels had no correlation to age, distal- or lymph node- metastasis, and clinical stage. Furthermore, there was no significant difference of *SIM2* mRNA expression between Stage IB1/IIA1 (tumor size <4 cm) and Stage IB2/IIA2 (tumor size >4 cm), indicating that SIM2 contributes not to tumor growth. In contrast, high *SIM2* mRNA expression significantly correlated to a good response to primary therapy. Since there are two splicing isoforms of *SIM2* mRNA (*SIM2s* and *SIM2l*) and their biological function is different as mentioned above, we validated which isoform (or both) affects the radio-sensitivity in CvSCC patients. Using another GEO database, expression levels of *SIM2s* or *SIM2l* between pre- and post-radiotherapy were compared in 20 or 18 patients, respectively. The expression level of S*IM2l* was significantly decreased after radiotherapy, while that of *SIM2s* was increased at post-therapy (Fig. 1d and Supplementary Figure S1d). These data suggested that SIM2l-expressed cancer cells can be selectively eradicated by radiotherapy, thereby residual tumor mainly consisted of cancer cells with no/low SIM2l expression. Therefore, a SIM2l-expressed tumor is sensitive to radiotherapy in CvSCC patients and becomes a good prognostic factor. Since all of these analyses were focused on *SIM2* mRNA, relationship between SIM2 protein expression and clinical outcome remains unclear. However, there was some correlation between mRNA and protein expression levels in CvSCC cell lines (Supplementary Figure S1e), suggesting SIM2 protein expression level also reflects survival and sensitivity to radiotherapy in CvSCC patients.

**Table 1:** Relationship between *SIM2* mRNA expression and clinicopathological features in cervical squamous cell carcinoma patients.

Variables	Number of cases	SIM2 mRNA*	*P*-value
Age (years)<			0.433
<50	146	182.4	
≥50	107	203.3	
Histological type			0.727
Keratinizing	55	193.6	
Non-keratinizing	114	181.0	
Lymph node status			0.171
No metastasis	104	208.7	
Metastasis	52	166.4	
Distal metastasis			0.899
No metastasis	33	179.8	
Metastasis	18	184.7	
Primary therapy outcome			0.0061
CR/PR	74	223.0	
PD	14	105.7	
TNM staging			0.656
Stage Ι	125	197.3	
Stage ΙΙ-ΙV	121	185.0	

*Average value.

### Loss of SIM2 upregulates HIF-1α and contributes to apoptosis resistance under hypoxia

**Figure 2:**
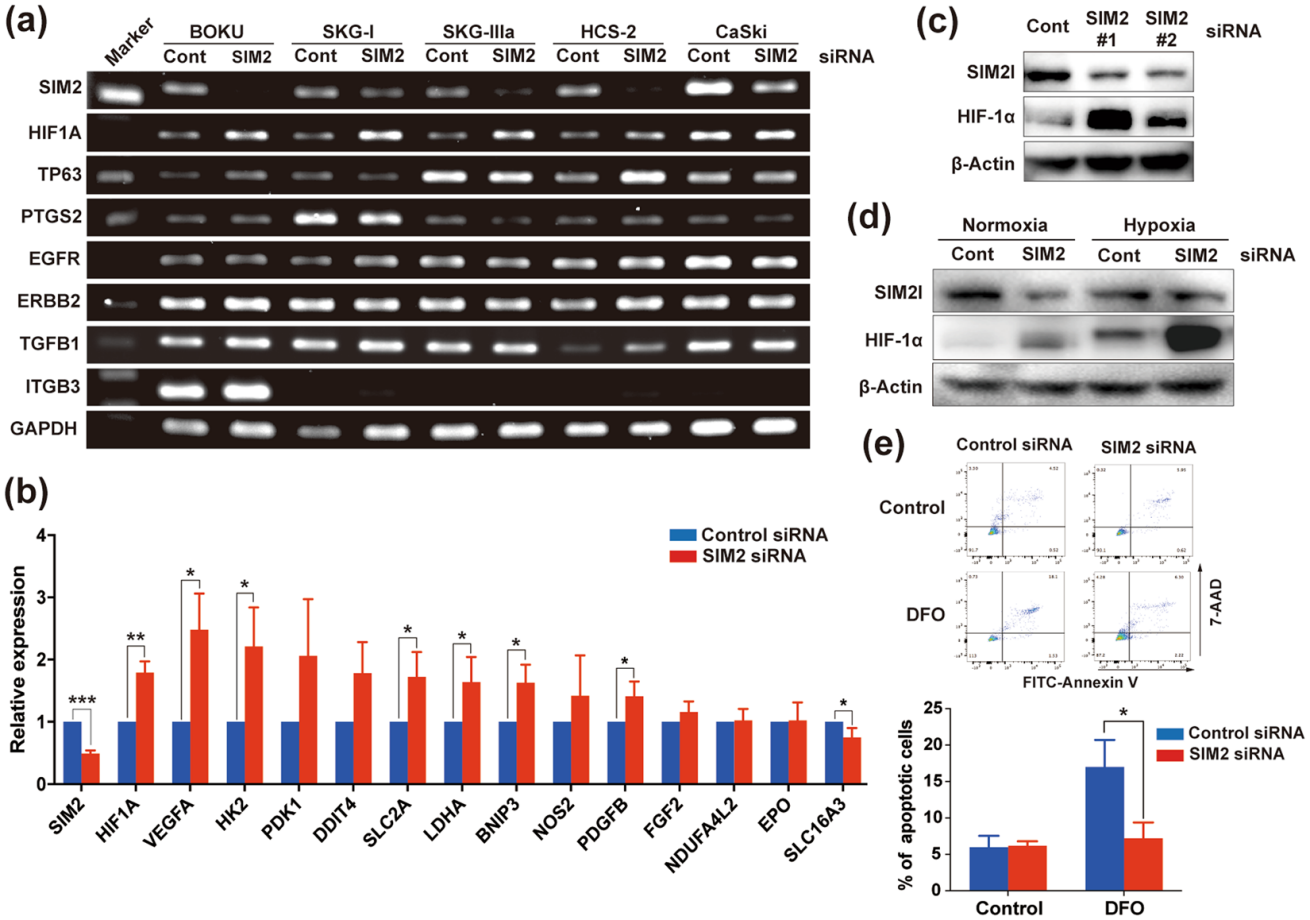
SIM2 knockdown induces HIF-1α and resistance to hypoxia. **(a)** RT-PCR analyses of radiosensitive genes in 5 CvSCC cell lines treated with control siRNA (cont) or *SIM2* siRNA (SIM2). *GAPDH* was used as a loading control. The cropped gels are used in the figure and full gels are presented in Supplementary Figure S2. **(b)** Quantitative RT-PCR (qRT-PCR) analyses of *HIF1A* and HIF-1α-target genes in control siRNA- or *SIM2* siRNA-treated SKG-ΙΙΙa cells. All data were obtained from 3 independent experiments and presented as mean ± SD. **(c)** Western blot analyses of SIM2l and HIF-1α in SIM2-knockdown SKG-ΙΙΙa cells. Two kinds of siRNAs (#1 and #2) were used for the analyses. β-Actin was used as a loading control. The cropped blots are used in the figure and full blots are presented in Supplementary Figure S3. **(d)** Induction of HIF-1α expression by hypoxia. SIM2l and HIF-1α expression was detected in SIM2-knockdown cells cultured under normoxia or hypoxia. β-Actin was used as a loading control. The cropped blots are used in the figure and full blots are presented in Supplementary Figure S5. **(e)** Apoptosis induction by hypoxia mimetic agent, deferoxamine treatment. SIM2-knockdown cells were treated by DMSO (Control) or 100 μM deferoxamine (DFO) for 24 hours. Apoptotic cells were detected by FITC-Annexin V and 7-AAD double staining followed by FACS analysis. Representative FACS data are shown at upper panels. A bottom panel shows the rate of apoptotic cells calculated by the sum of early apoptotic fraction (AnnexinV^High^ and 7-AAD^Low^) and late apoptotic one (AnnexinV^High^ and 7-AAD^High^). Data were obtained from 3 independent experiments and presented as mean ± SD. All statistical analyses were performed by student t-test. *, **, and *** represent *p* < 0.05, *p* < 0.01, and *p* < 0.001, respectively.

We then searched SIM2-target genes in CvSCC, because SIM2 has been reported to act as a transcriptional repressor. Using siRNA-based transient knockdown of SIM2 in five CvSCC cell lines (BOKU, SKG-Ι, SKG-ΙΙΙa, HCS-2, and CaSki), we searched the target from 7 candidate genes (*HIF1A*, *TP63*, *PTGS2*, *EGFR*, *ERBB2*, *TGFB1*, *ITGB3*) which have been reported as markers sensitive to radiotherapy in CvSCC^27^. By semi-quantitative RT-PCR analysis, *HIF1A* was only a gene in which mRNA expression was increased by SIM2 knockdown in more than one CvSCC cell line (Fig. 2a and Supplementary Figure S2). Among 6 CvSCC cell lines, we used 2 cell lines (SKG-Ι and SKG-ΙΙΙa) for following analyses, because *HIF1A* expression was highly induced by SIM2 knockdown. In SKG-ΙΙΙa, quantitative real-time RT-PCR (qRT-PCR) revealed that SIM2 knockdown resulted in an increase of not only *HIF1A* but also its direct target genes including *VEGFA*, *HK2*, *SLC2A1*, *LDHA*, *BNIP3*, and *PDGFB* (Fig. 2b), indicating that SIM2 controls these genes through suppression of *HIF1A* expression. Consistent with these observations, expression of HIF-1α was increased by SIM2 knockdown (Fig. 2c, Supplementary Figures S3, S4a and S4b). Next, we tested whether increased expression of HIF-1α by SIM2 knockdown is a consequent of upregulation of four genes (*EGLN1*, *EGLN2*, *EGLN3*, and *HIF1AN*) that are known to degradate HIF-1α protein. The mRNA expression levels of these genes were not strongly affected by SIM2 knockdown (Supplementary Figure S4c), indicating that SIM2 directly suppresses *HIF1A* expression. It is well-known that HIF-1α is dramatically increased under hypoxia and contributes to apoptosis resistance. Therefore, we examined HIF-1α expression by SIM2 knockdown under both normoxia and hypoxia, which showed that, under hypoxia, HIF-1α was notably increased (Fig. 2d and Supplementary Figure S5). SIM2 knockdown also significantly reduced apoptotic cells by treatment of deferoxamine (DFO), which can mimic hypoxia, whereas the knockdown did not affect apoptosis under normoxia (Fig. 2e). These results suggest that SIM2 acts as a transcriptional suppressor to *HIF1A* and contributes to the escape from apoptosis of CvSCC cells under hypoxic stress.

### SIM2 loss enhances cell growth and resistance to oxidative and radiation stresses under 3D environment

**Figure 3:**
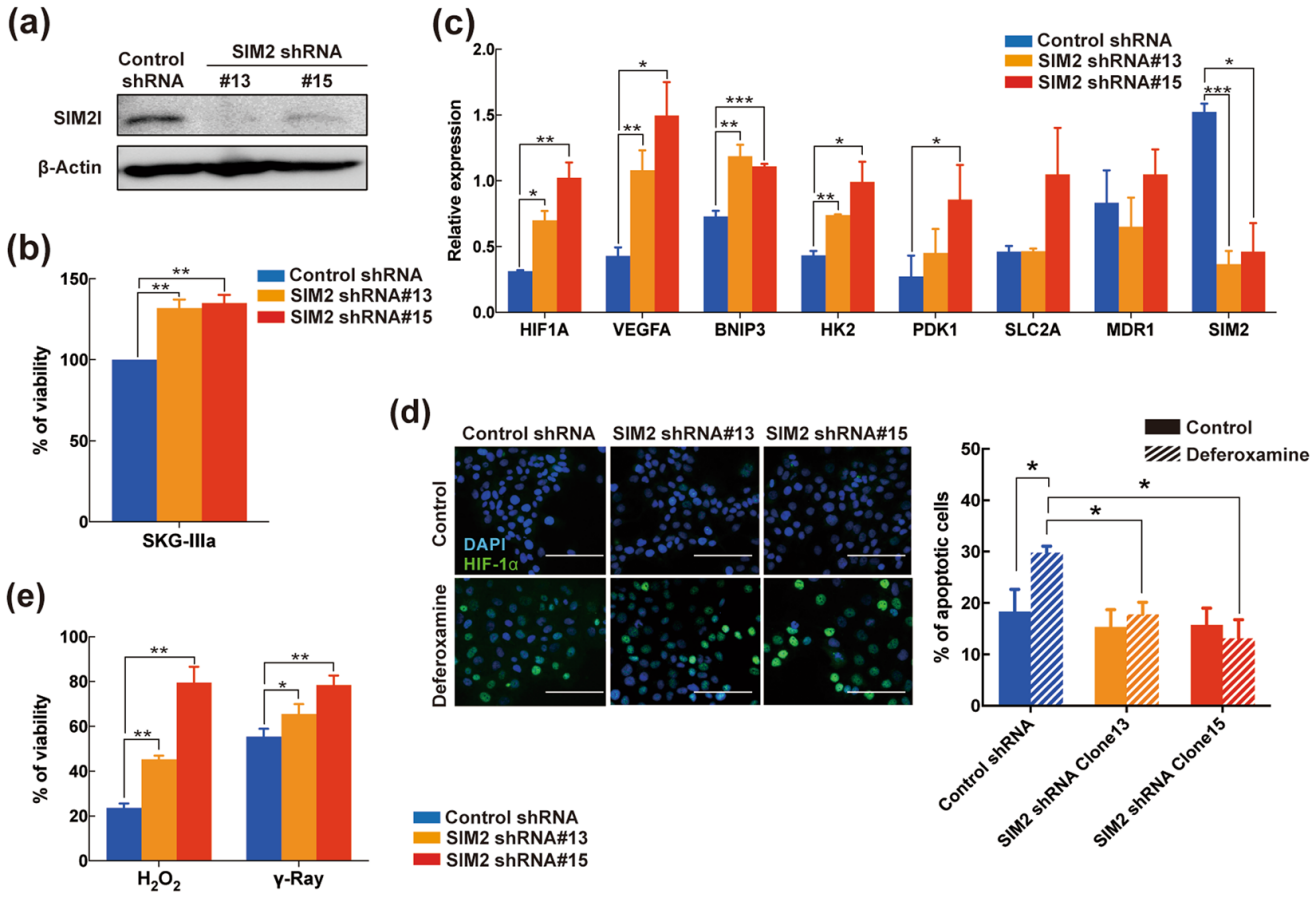
SIM2 knockdown enhances survival and resistance to radiation in a 3D culture. (**a**) Western blot analysis of SIM2l in *SIM2* shRNA-expressing SKG-ΙΙΙa cells. Two knockdown clones (#13 and #15) were established and analyzed. β-Actin was used as a loading control. The cropped blots are used in the figure and full blots are presented in Supplementary Figure S6. **(b)** Cell viability assay of SIM2-knockdown cells cultured for 9 days in a 3D plate. Each viability was normalized by control shRNA-expressing cells. **(c)** qRT-PCR analysis of *HIF1A* and its target genes (*VEGFA*, *BNIP3*, *HK2*, *PDK1*, *SLC2A*, and *MDR1*) in SIM2-knockdown cells under a 3D culture. **(d)** SIM2 knockdown induces HIF-1α and resistance to apoptosis. Left panels show representative fluorescent images of immunohistochemistry for HIF-1α (Green) after 24-hour exposure by DMSO control (top panels) or 100 μM deferoxamine (bottom panels). All images were overlaid by DAPI staining for detection of nuclei (blue). Each bar represents 100 μm. Right panels show apoptosis detection of SIM2-knockdown cells treated by DMSO (solid bar) or 100 μM deferoxamine (striped bar) for 24 hours. Calculation of apoptotic fractions is described elsewhere. **(e)** Sensitivity of SIM2-knockdown cells against H_2_O_2_ and γ-ray. Cells were cultured under 3D condition followed by 24-hour exposure of 1 mM H_2_O_2_ or 5 Gy of γ-ray irradiation. Data were normalized by untreated control. Data were obtained from 3 independent experiments and presented as mean ± SD. All statistical analyses were performed by student t-test. *, **, and *** represent p < 0.05, p < 0.01, and p < 0.001, respectively.

As demonstrated above, an anti-apoptotic effect by SIM2 knockdown was detected only under hypoxia even though HIF-1α was upregulated under both normoxia and hypoxia. This finding suggested that SIM2 loss did not affect a cancer phenotype under normoxia. On the other hand, actual tumors are exposed by various stresses including ROS, low-level oxygen availability, and under-nutrition, all of which cannot accurately be recapitulated by a 2D plate culture. In this regard, we validated various cancer phenotypes under a long-term 3D culture, which somewhat mimics such tumor microenvironments as cell-cell interaction and hypoxia. To validate the SIM2-knockdown effect under a 3D culture, we first established two *SIM2* shRNA-expressing SKG-ΙΙΙa clones (#13 and #15) (Fig. 3a and Supplementary Figure S6). Although there was no difference between control shRNA-expressing mixed clones and the two *SIM2* shRNA-expressing clones under a 2D culture (Supplementary Figure S7a), both of the two SIM2-knockdown clones significantly promoted spheroid growth in low-adhesive 3D culture plates (Fig. 3b). Furthermore, the spheroid formation ability of SIM2-knockdown clones was significantly higher than that of the control mixed clones (Supplementary Figure S7b), suggesting that SIM2-knockdown CvSCC cells have an advantage of growth promotion *in vivo*. Notably, induction of *HIF1A* mRNA by SIM2 knockdown was strongly enhanced under the 3D culture compared to the 2D culture (Fig. 3c). On the other hand, SIM2 knockdown did not induce *MDR1* which have been reported to a key drug resistant gene regulated by HIF-1α^28^. Consistent with our results of the transient *SIM2-*siRNA transfection, stable SIM2-knockdown clones suppressed hypoxia-induced apoptosis under a hypoxia mimic condition, and immunocytology showed that HIF-1α induction was higher in the SIM2-knockdown clones than in the control shRNA-expressing mixed clones (Fig. 3d). Next, we investigated whether sensitivity to a cytotoxic drug, cisplatin (CDDP), oxidative stress, or radiation is decreased by SIM2 knockdown, because the CvSCC patients with low SIM2 expression showed a worse prognosis for both chemoradiotherapy and radiotherapy (Fig. 1c and Supplementary Figure S1c). Although there was no difference in the sensitivities to CDDP between control shRNA-expressing mixed clones and SIM2-knockdown clones under a 3D culture (Supplementary Figure S3c), the SIM2-knockdown clones showed increased resistance to both H_2_O_2_ and γ-ray treatment (Fig. 3d). These data supported our hypothesis that loss of SIM2 protects oxidative and radiation stress, resulting in a poor outcome for irradiation-based therapies in CvSCC patients.

### SIM2l acts as a tumor suppressor gene *via* suppression of angiogenesis and induction of apoptosis *in vivo*

**Figure 4:**
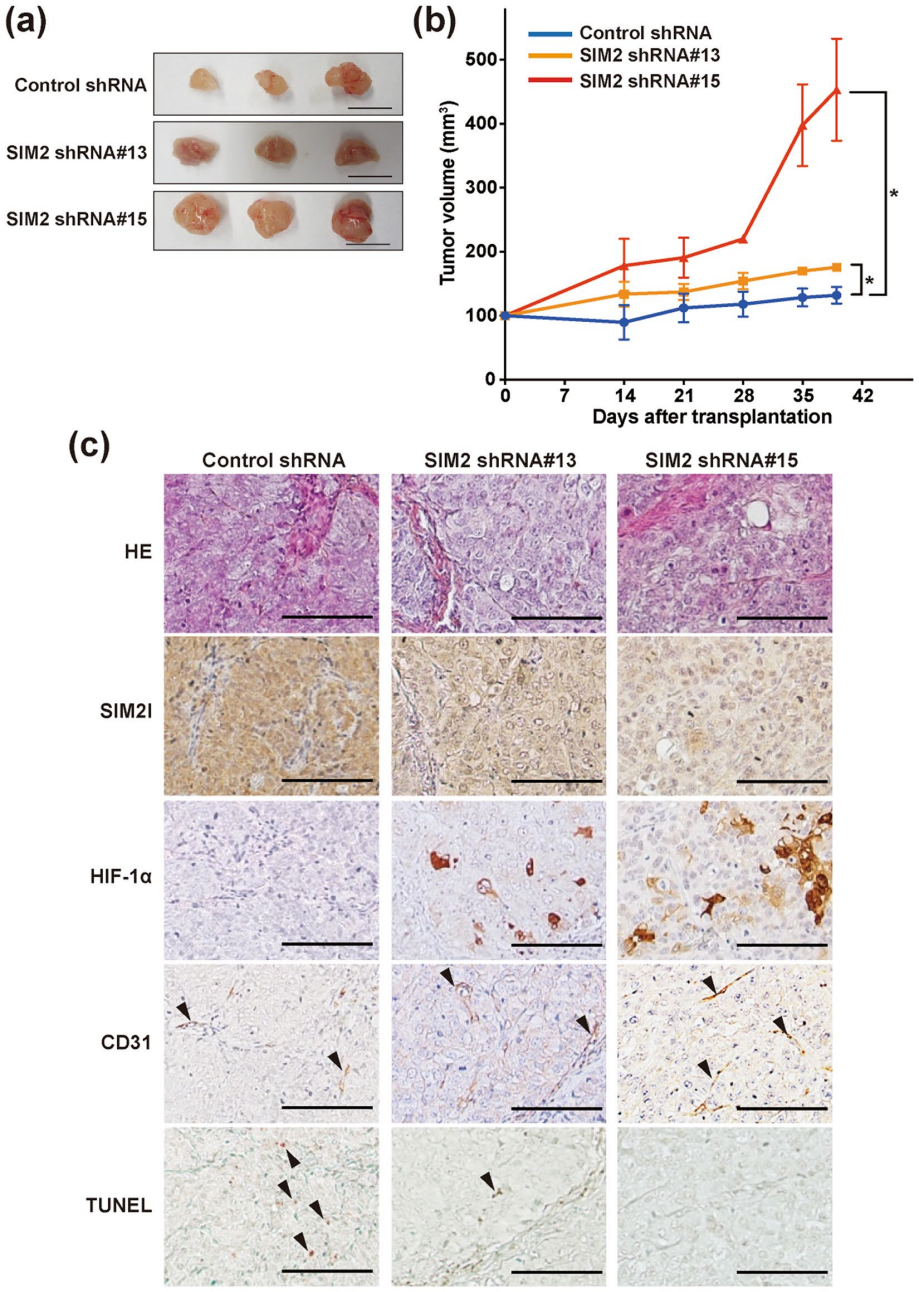
SIM2 knockdown enhances tumor growth *in vivo*. **(a)** Images of tumors subcutaneously grafted by control shRNA- and *SIM2* shRNA-expressing SKG-ΙΙΙa cell lines. Each bar represents 1 cm. **(b)** Tumor progression of SIM2-knockdown cell lines. Tumor size was measured weekly after 2 weeks from graft. Data were obtained from 3 mice and presented as mean ± SD. Statistical analyses were performed by student t-test. *Represents *p* < 0.05. **(c)** Representative immunohistochemical staining for SIM2l, HIF-1α, CD31, and TUNEL of xenografted tumors of SIM2-knockdown cell lines. Each bar represents 100 μm. The magnified pictures are used in the figure and full ones are presented in Supplementary Figure S9.

**Figure 5:**
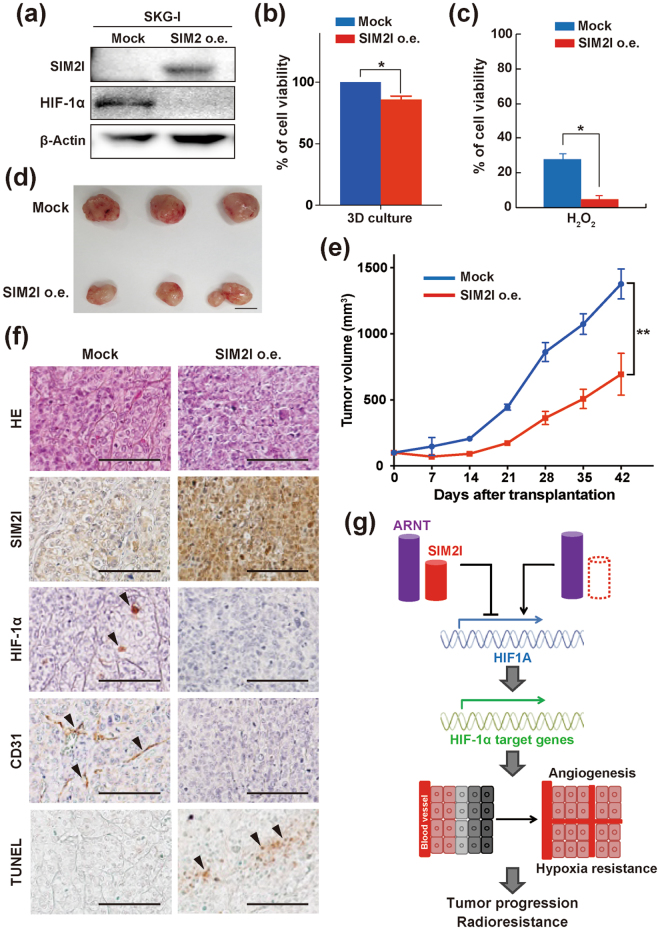
SIM2l suppresses tumor growth *in vitro* and *in vivo*. **(a)** Western blot analysis for SIM2l and HIF-1α in mock vector transfected- and SIM2l-overexpressing SKG-ΙΙΙa cell line (Mock and SIM2l o.e., respectively). β-Actin was used as a loading control. The cropped blots are used in the figure and full blots are presented in Supplementary Figure S8. **(b)** Cell viability measurement of SIM2l-overexpressing cells at 9 days under a 3D culture. Each data is normalized by mock transfectants. **(c)** Sensitivity to H_2_O_2_ treatment under a 3D culture. Mock and SIM2l-overexpressing cells were cultured under 3D condition followed by 500 μM H_2_O_2_ exposure for 24 hours. Each data is normalized by the viability of untreated control. Statistical analyses were performed by student t-test. *Represents *p* < 0.05. **(d)** Images of tumors subcutaneously grafted by mock and SIM2l-overexpressing cell lines. **(e)** Progression of tumor xenografted by SIM2l-overexpressing cells. Tumor size was measured weekly after a week from graft. Data were obtained from 3 mice and presented as mean ± SD. Statistical analyses were performed by student t-test. *Represents *p* < 0.05. **(f)** Representative immunohistochemical staining for SIM2l, HIF-1α, CD31, and TUNEL of xenografted tumors of SIM2l-overexpressing cells. Each bar represents 100 μm. The magnified pictures are used in the figure and full ones are presented in Supplementary Figure S10. **(g)** Schematic diagram of the roles of SIM2l as a suppressor to tumor progression and radio-resistance in the context of HIF-1α regulation. Details are described in the text.

We also verified whether our findings from the above *in vitro* studies are also observed in a mouse xenograft model. SIM2-knockdown SKG-ΙΙΙa cell lines (*SIM2* shRNA #13 and #15) were subcutaneously grafted to immunodeficient mice and monitored for tumor progression. Consistent with the *in vitro* studies, tumor growth of the SIM2-knockdown clones was significantly higher than that of the control shRNA clones (Fig. 4a and b, and Supplementary Table S1). Immunohistochemical analysis revealed that xenografted tumors of SIM2-knockdown clones increased HIF-1α expression (Fig. 4c and Supplementary Figure S9) and showed induction of angiogenesis (CD31 staining in Fig. 4c) and suppression of apoptosis (TUNEL staining in Fig. 4c). As shown in Fig. 1d and Supplementary Figure S1e, gene expression profiles from microarray database (Genome Expression Omnibus) indicated that overexpression of only a long isoform SIM2l is associated with radio-sensitivity in CvSCC patients. Therefore, we prepared SIM2l-overexpressing cells consisting of cDNA transfected-SKG-I mixed clones (SIM2 o.e.) (Fig. 5a and Supplementary Figure S8) and validated their functions from various aspects. Here, too, as in the observations in SIM2-knockdown experiments, cell growth ability under normoxia and the chemosensitivity of SIM2l-overexpressing cells were similar to those of mock-vector transfected cells (Supplementary Figure S7d and e, respectively). In contrast, SIM2l overexpression resulted in suppression of spheroid growth and in increased sensitivities to H_2_O_2_ and γ-ray (Supplementary Figure S7b and c, respectively). Furthermore, SIM2l-overexpressing cells significantly suppressed tumor growth in a mouse subcutaneous xenograft model (Fig. 5d and e, and Supplementary Table S2). Immunohistochemical analyses also revealed that xenografted tumors of SIM2l-overexpressing cells showed low angiogenesis with decreased HIF-1α expression and also showed increased apoptosis (Fig. 5f and Supplementary Figure S10). These data strongly indicate that SIM2l independently acts as a tumor suppressor gene *via* suppression of HIF-1α-induced angiogenesis and of hypoxia resistance in CvSCC patients who received radiotherapy (Fig. 5g).

## Discussion

Although chemoradiotherapy is an important modality for CvSCC, the relapse rate has been reported to be limited (28–64% in FIGO stages IIb–IVa)^28^. Therefore, predicting the response to chemoradiotherapy and presenting therapeutic options for a poor response group are of utmost significance for patients. Great efforts to identify such predictive biomarkers have been made by many researchers. However, only a few biological variances exist for cervical cancer patients who received chemoradiotherapy^29^. In this study, we demonstrate that SIM2 has a potential to be an independent prognostic marker for CvSCC patients. High SIM2 expression was positively correlated to OS and response to radiotherapy in CvSCC patients (Fig. 1c and d). It has been reported that SIM2 dimerizes with ARNT, binds to CME, and transcriptionally represses expression of target genes. A recent ChIP-sequencing study reported that 22 genes (*e*.*g*. *Otx2*, *Arid1b*, and *Syngr1*) are candidates for a mouse Sim2 target in embryonic stem cells^30^. However, little is known about its direct target genes in human cells including cancer cells. Since *SIM2l* mRNA expression was decreased after treatment of radiotherapy (Fig. 1d), we hypothesized that SIM2 can downregulate genes that are involved in radio-resistance. From currently-reported radio-resistant genes in cervical cancer^27^, we identified *HIF1A* encoding HIF-1α as a target by SIM2 (Fig. 2a and Supplementary Figure S4a). As with SIM2, HIF-1α is a bHLH-PAS family member and activates more than 100 genes related to hypoxic response (*i*.*e*., anti-apoptosis, angiogenesis, metabolism, and proliferation) upon binding to ARNT^31^. It is well-known that SIM2 can indirectly suppress HIF-1α function through deprivation of ARNT^32^. However, no report about the direct relationship between SIM2 and *HIF1A* mRNA expression exists. Here, we provided the first evidence that SIM2 represses transcription of *HIF1A*. Notably, tumor hypoxia has been reported to have a major impact on the outcome of definitive radiotherapy and chemoradiotherapy among these variances, since its niche is thought to be an abolished oxygen enhancement effect^29,33^. Especially, increased expression of HIF-1α and its targets (*e*.*g*., vascular endothelial growth factor, hexokinases 2, and GLUT-1) have been reported to correlate with a worse prognosis in cervical cancer patients who received radiotherapy or chemoradiotherapy^34–37^. Considering that HIF-1α is an important transcriptional factor in adapting to severe hypoxia (*e*.*g*. cell cycle arrest, anti-apoptosis, and angiogenesis), it can also be a key contributor to radio resistance^38^. As expected, knockdown of SIM2 increased not only *HIF1A* but also its target genes (*i*.*e*. *VEGFA*, *HK2*, and *BNIP3*) (Fig. 2b). In consistent with the result that SIM2 knockdown did not contribute to chemoresistance (Supplementary Figure S7c), it did not alter *MDR1* expression (Fig. 3c). Therefore, HIF-1α-MDR1 transcriptional pathway may be not involved in CDDP resistance. Although SIM2 knockdown induced *HIF1A* expression, SIM2 did not suppress activity of the −1311 to +281 *HIF1A* promoter (not shown). Identification of the distal promoter is needed to reveal the mechanism of SIM2-mediated *HIF1A* downregulation. In addition to resistance to hypoxia in normal culture, SIM2 knockdown significantly promoted cell proliferation and spheroid formation under a 3D culture (Figs 2e and 3b, and Supplementary Figure S7b), which can mimic hypoxia^39^. Despite SIM2 knockdown induced *HIF1A* expression, it did not promote cell growth under normoxic 2D culture. These facts may be attributed to rapid degradation of HIF-1α by the ubiquitin-proteasome under normoxia. We also found that SIM2 knockdown reduced sensitivity to both oxidative stress and radiation under a 3D culture (Fig. 3e). These results are supported by previous reports that HIF-1α attenuates radio sensitivity and oxidative stress^40–42^. In agreement with those observations in a 3D culture, SIM2 knockdown promoted tumor growth with enhancement of HIF-1α expression and angiogenesis *in vivo* (Fig. 4). These results also underscored that SIM2^Low^ CvSCC patients face a poor prognosis (Fig. 1c) and more importantly indicate that targeting angiogenesis (*i*.*e*., chemotherapy by anti-VEGF antibody, Bevacizumab) may be a good therapeutic strategy for them. Moreover, our meta-analyses indicated that radio-sensitivity is ascribed not to the SIM2 short isoform but to the long one (Fig. 1d and Supplementary Figure S1d). SIM2l-overexpressing CvSCC cells showed both cell growth inhibition and sensitivity to ROS in a 3D culture, and SIM2l-overexpressing tumors suppressed HIF-1α expression, angiogenesis, and tumor progression *in vivo* (Fig. 5b,c,e and f). Although SIM2l overexpression suppressed HIF-1α (Fig. 5a), both suppression of angiogenesis and induction of apoptosis was not observed *in vitro* (not shown). The inconsistent result between *in vitro* and *in vivo* may be attributed to functional change of HIF-1α depending on environment (e.g. hypoxia). Therefore, it is of important to reveal what kind of tumor niche contributes to enhancement of the SIM2l-HIF-1α axis in CvSCC.

In conclusion, SIM2l attenuates HIF-1α-mediated hypoxia- and radio-resistance; thus it has a potential not only as a radio-sensitive marker for CvSCC patients but as a way to provide a new therapeutic strategy for an SIM2 negative-radio-resistant one.

## Materials and Methods

### External data analysis

*SIM2* mRNA expression data of 309 cervical cancer patients was downloaded as z-scores (RNA Seq V2 RSEM) from the cBioPortal (http://www.cbioportal.org/). The full clinical dataset, including age, gender, histological type, disease stage, treatment history, and overall survival status/period, were also downloaded from the TCGA portal and linked with genetic data. After excluding samples which histological types are not squamous cell carcinoma (n = 256), overall survival of SIM2^High^ and SIM2^Low^ quadrant group was analyzed using the Kaplan-Meier method, and differences between two survival curves were tested by the log-rank test in the different groups. *SIM2s* and *SIM2l* mRNA expression data was also downloaded from ArrayExpress (GEOD-27678, https://www.ebi.ac.uk/arrayexpress/) which dataset consists of cervical cancer biopsy samples from patients before/after receiving radiotherapy or CRT.

### Cell lines

Human cervical cancer cell lines (BOKU, SKG-I, SKG-IIIa, HCS-2, and CaSKi) were purchased from the Japanese Collection of Research Bioresources Cell Bank. BOKU, SKG-I, SKG-IIIa were cultured in Ham’s F12 (Wako, Tokyo, Japan) with 10% of fetal bovine serum (FBS) and 100 U/ml of penicillin-streptomycin. HCS-2 was cultured in EMEM (Wako, Tokyo, Japan) with 15% of bovine serum (FBS) and 100 U/ml of penicillin-streptomycin. CaSki was cultured in RPMI 1640 (Wako, Tokyo, Japan) with 10% of bovine serum (FBS) and 100 U/ml of penicillin-streptomycin.

### siRNA transfection

In all experiments, we performed siRNA transfection under the same condition of 75 pmol siRNA/2 × 10^5^ cells at final concentration of 75 nM. Cells were transfected with control siRNA (#AM4611; Ambion, Austin, TX, USA) and *SIM2* siRNA (#s12869, s12868; Ambion, Austin, TX, USA) using Thermo Scientific DharmaFECT Transfection Reagents (Thermo Fisher Scientific, MA, USA) following the procedure recommended by the manufacturer. At 48 or 72 hours after siRNA transfection, cells were analyzed by RT-PCR or western blotting, respectively. At 24 hours after siRNA transfection, cells were exposed to normoxia (21% O_2_ and 5% CO_2_) or hypoxia (1% O_2_ and 5% CO_2_) for 48 hours.

### RT-PCR and quantitative real-time RT-PCR

Total RNA was isolated from cells in an ISOGEN lysis buffer (Nippon Gene, Toyama, Japan) followed by precipitation with isopropanol. Reverse transcription was carried out by SuperScript III First-Stand Synthesis System for RT-PCR (Invitrogen, CA, USA). PCR was carried out by AccuPrimeTaq DNA Polymerase System (Invitrogen, CA, USA). RT-PCR was performed within the linear range of amplification, typically 19–30 cycles, for *SIM2*, *HIF1A*, *TP63*, *PTGS2*, *EGFR*, *ERBB2*, *TGFB1*, *ITGB3*, and *GAPDH*. Quantitative real-time PCR was performed on *HIF1A*, *VEGFA*, *HK2*, *PDK1*, *DDIT4*, *SLC2A*, *LDHA*, *BNIP3*, *NOS2*, *PDGFB*, *FGF2*, *NDUFA4L2*, *EPO*, *SLC16A3*, *MDR1*, *SIM2*, and *GAPDH* by a Bio-Rad iCycler with iQSyber Green Supermix (Bio-Rad, Hercules, CA, USA) according to the manufacturer’s instructions. DNA sequences of used primers are listed in Supplementary Table S3.

### Western blotting

Cultured cells were lysed by 1 × Laemmli Sample Buffer (Bio-Rad Laboratories) containing 350 mM DTT (Thermo Fisher Scientific) and 1% Protease Inhibitor Cocktail (Sigma-Aldrich). Samples were electrophoresed by NovexWedgeWell 4–20% Tris-Glycine Gel (Thermo Fisher Scientific) and transferred to Immobilon-P PVDF membranes (Merck Millipore, Massachusetts, USA). After blocking with 5% of Membrane Blocking Agent (GE Healthcare, Buckinghamshire, UK) in PBS, membranes were probed with optimal concentration of primary antibodies overnight at 4 °C. Primary antibodies included: goat polyclonal anti-SIM2l (#sc-8716, Santa Cruz Biotechnology, Santa Cruz, CA, USA, 1:200), rabbit polyclonal anti-HIF1 (#14179s, Cell Signaling Technology, 1:1000) and mouse monoclonal anti-β-Actin (#4967, Cell Signaling Technology, 1:1000). After washing with Tris-buffered saline and Tween 20, membranes were incubated with correspondent secondary antibodies. Secondary antibody included: HRP-conjugated goat anti-mouse antibody (#P0449, DAKO, Carpinteria, CA), 1:2000), HRP-conjugated rabbit anti-mouse antibody (#P0399, DAKO, Carpinteria, CA, 1:3000) and HRP-conjugated mouse anti-mouse antibody (#P0260, DAKO, Carpinteria, CA, 1:1000). After washing, membranes were incubated with Pierce ECL Plus Western Blotting Substrate (Thermo Scientific, IL, USA) and chemiluminescent signals were detected by ImageQuant LAS 4000 mini system (GE Healthcare).

### shRNA-based gene knockdown

2.5 × 10^4^ cells were seeded in a 24-well plate and incubated at 37 °C overnight. The cells were incubated in a serum free medium containing 5 μg/ml polybrene (Santa Cruz Biochemistry) at 37 °C for 4 hours. After incubation, 5 × 10^3^ or 2.5 × 10^4^ IFU of control and human *SIM2* shRNA lentiviral particles (Santa Cruz Biochemistry) were added to the cells respectively and cultured in a penicillin-streptomycin free medium at 37 °C overnight. The medium with lentiviral particles was removed and incubated in normal medium for 2 days. The shRNA-expressing cells were selected by culture with selection medium containing 0.5 μg/ml puromycin (Thermo Fisher Scientific). The cells were isolated into single cell and cultured in a 96-well plate to obtain clone cells. For quantitative real-time RT-PCR, 3 × 10^6^ cells were seeded per 3.5 cm EZSPHERE Dish (IWAKI, Chiba, Japan) and incubated for 9 days.

### Plasmid construction and transfection

2.5 × 10^4^ cells were seeded in a 24-well plate and incubated at 37 °C overnight. The cells were incubated in serum free medium containing 5 μg/ml polybrene (Santa Cruz Biochemistry) at 37 °C for 4 hours. After incubation, cells were transfected with 1 μg of pCDH-CMV-mock or pCDH-CMV-SIM2l and cultured in a penicillin-streptomycin free medium at 37 °C overnight. The medium was removed and incubated in normal medium for 2 days. The cells were selected by culture with selection medium containing 0.5 μg/ml puromycin (Thermo Fisher Scientific).

### Cell viability assays

2D culture system: cells were seeded at 1 × 10^5^ cells per well in 6-well plates. After 24 hours or 48 hours of incubation, cells were trypsinized and the viable cells were counted every day.

3D culture system: cells were seeded at 2 × 10^4^ cells per well in a 96-well EZSPHERE plate (IWAKI, Chiba, Japan) and incubated for 9 days to estimate 3D viability. To estimate H_2_O_2_ and γ-ray sensitivity, cells were incubated for 2 days before exposure. For estimating H_2_O_2_ sensitivity, cells were exposed to H_2_O_2_ (0, 500 μM, 1 mM) for 24 hours. For estimating γ-ray sensitivity, cells were irradiated with γ-ray (0, 5Gy) and incubated for 7 days. Viable cells were detected by CellTiter-Glo 3D Cell Viability assay (Promega, Madison, WI, USA) according to the manufacturer’s instructions.

### Colony formation assay

Pre-chilled 6-well plates were coated with 500 μl of Matrigel (BD Bioscince) per well and incubated at 37 °C for 30 min. 2 × 10^5^ cells were pelleted by centrifugation, resuspended into 1 ml of medium and plated onto the coated surface. After mixing medium and 10% of Matrigel (BD Bioscince), 1ml of Matrigel-medium mixture was added to the plated culture. Cells were cultured for 96 hours, and the areas of spheres were quantified by ImageJ. Experiments were performed according to the protocol published by Bissell^43^.

### Immunofluorescence analysis

Cells were cultured on glass chamber slides at 37 °C overnight and exposed to deferoxamine (0, 100 μM) for 24 hours. Cells were then fixed with 4% paraformaldehyde for 15 minutes, permeabilized with −20 °C methanol and 0.5% Triton X-100/PBS, and blocked with 10% fetal bovine serum and 2% bovine serum albumin in PBS. Cells were incubated with primary antibody for HIF-1α (#ab51608, Abcam, Cambridge, UK, 1:500) at 4 °C overnight. After washing by PBS twice, cells were incubated with Alexa 488-conjugated anti-rabbit IgG antibody (Invitrogen, CA, USA, 1:200) at room temperature for 10 minutes. After washing by PBS twice, the nuclei were stained with DAPI.

### Mouse xenograft model

Six-week-old female BALB/c-nu/nu mice were purchased from Charles River Laboratories (Beijing, China) and bred at a room temperature with a 12 hours’ light/dark daily cycle. The mice were maintained under specific pathogen-free conditions and provided sterile food, water, and cages. 3 × 10^6^ or 6 × 10^6^ of cancer cells were suspended in a 3:2 mixture of PBS and Matrigel (BD bioscience) and then transplanted subcutaneously in the back of the mice by use of a 26 1/2-gauge needle. Body weight and tumor volume of the mice were measured weekly. Tumor volume was calculated using the following formula: tumor volume = D/2 × (d/2)^2^ × 4/3π, in which D and d refer to the long and short tumor diameter. The mice were euthanized by anesthetic overdose at 6 weeks after the transplantation. We did not observe any pain behaviors and symptoms (*e*.*g*. impaired mobility, anemia, severe weight loss, or excess tumor growth) in all mice during the period. All experiments were conducted in accordance with the ethical guidelines of the International Association for the Study of Pain and were approved by the Committee for Ethics in Animal Experimentation of the National Cancer Center. Efforts were made to minimize the numbers and any suffering of animals used in the experiments.

### Patients’ samples

The tissues of cervical squamous cell carcinoma and normal cervix were obtained from patients (4 and 2 cases, respectively) who underwent surgery at Keio University Hospital (Tokyo, Japan). All patients provided written, informed consent, and the study protocol (No. 2007-0081) was approved by the ethics committee of Keio University. Experiments with these samples were performed in accordance with the approved guidelines.

### Immunohistochemistry

Tissues were fixed in formalin and embedded in paraffin. Tissue blocks were sliced into 4 μm sections. After deparaffinization and rehydration, antigen retrieval was performed by autoclaving at 90 °C for 30 minutes in Tris-EDTA buffer (pH 9.0). For staining CD31, antigen retrieval was performed by autoclaving at 90 °C for 30 minutes in 0.5 M Tris buffer. After blocking endogenous peroxidase activity, tissue sections were blocked by PBS with 10% fetal bovine serum for 30 minutes and stained with primary antibodies against SIM2l (#sc-8716, Santa Cruz, CA, USA, 1:50), HIF-1α (#ab51608, Abcam, Cambridge, UK, 1:50), and CD31 (#ab28364, Abcam, Cambridge, UK, 1:100). After incubation overnight at 4 °C, tissue sections were incubated with anti-goat secondary antibody for staining SIM2l and EnVision+ Dual Link System-HRP (Dako, Carpinteria, CA, USA) for staining other targets following coloring by DAB (Dako, Carpinteria, CA, USA). All samples were counterstained with Mayer hematoxylin. TUNEL staining was performed by *in situ* Apoptosis Detection Kit (TaKaRa, Shiga, Japan) according to the manufacturer’s instructions.

### Statistical analysis

All data were expressed as the mean ± SD obtained from 3 independent experiments and p-values were calculated using unpaired t-test. In clinical data, overall survival (OS) was estimated by the Kaplan-Meier method and p-values were calculated by log rank test using GraphPad Prism version7 (GraphPad Software, California, USA). Values of p < 0.05 were considered significant (*p < 0.05, **p < 0.01, and ***p < 0.001).

## Electronic supplementary material

Supplementary Information
