# Supplementary material for: SIM2l attenuates resistance to hypoxia and tumor growth by transcriptional suppression of HIF1A in uterine cervical squamous cell carcinoma
Source: Sci Rep. 2017 Nov 6;7:14574. doi: 10.1038/s41598-017-15261-4 (PMC5674005; doi:10.1038/s41598-017-15261-4)

**SIM2l　attenuates resistance to hypoxia　and tumor growth　by transcriptional suppression of　*HIF1A*in uterine cervical squamous cell carcinoma**

Kanako Nakamura^1^, Masayuki Komatsu^2*^, Fumiko Chiwaki^2^, Takashi Takeda^1^, Yusuke Kobayashi^1^, Kouji Banno^1^, Daisuke Aoki^1^, Teruhiko Yoshida^3^, and Hiroki Sasaki^2*^

^1^Department of Obstetrics and Gynecology, Keio University School of Medicine, Tokyo, Japan; ^2^Department of Translational Oncology, National Cancer Center Research Institute, Tokyo, Japan; ^3^Fundamental Innovative Oncology Core Center, National Cancer Center Research Institute, Tokyo, Japan.

***Correspondence:**

Hiroki Sasaki or Masayuki Komatsu

Department of Translational Oncology,

Fundamental Innovation Oncology Core Center,

National Cancer Center Research Institute

Tsukiji 5-1-1, Chuo-ku, Tokyo 104-0045, Japan

Phone: 81-3-3542-2511, ext. 3141

FAX: 81-3-3248-1631

E-mail: hksasaki@ncc.go.jp or makomats@ncc.go.jp

**Supplementary Figures and Tables**

**Supplementary Figure S1**

**
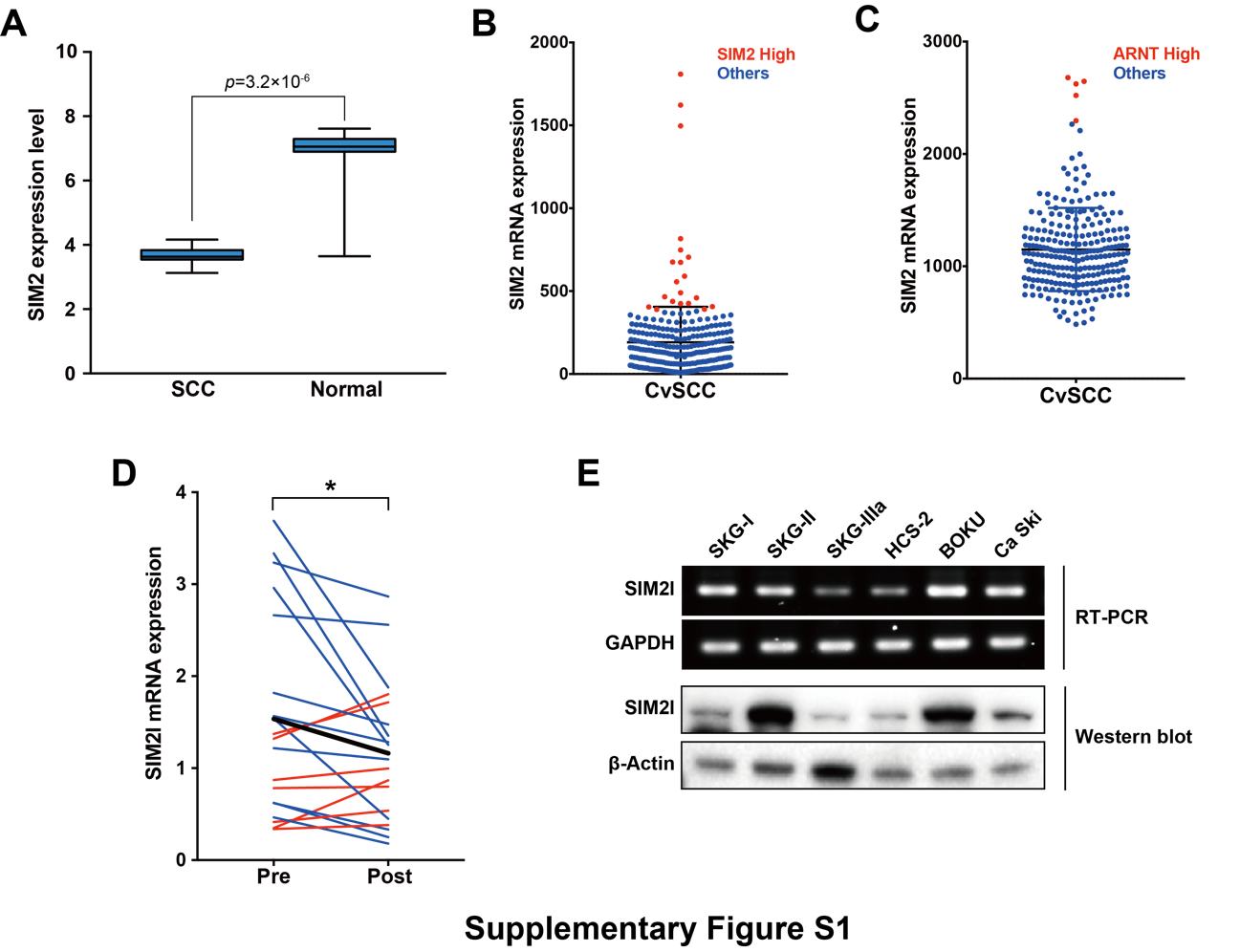
**

**Supplementary Figure S1. SIM2 and ARNT expression in CvSCC patients and cell lines.**

**(A)** *SIM2* mRNA expression of CvSCC (SCC, 28 cases) and normal cervix (Normal, 15 cases). Data was analyzed by student t-test. **(B) Distribution of *SIM2* mRNA expression among CvSCC (253 cases). Red dots indicate SIM2 highly-expressed cases (more than double the average).** **(C)** **Distribution of *ARNT* mRNA expression among CvSCC (253 cases). Red dots indicate SIM2 highly-expressed cases (more than double the average). (D)** Expressional change of *SIM2s* mRNA encoding a short isoform by radiotherapy in each CvSCC patient. Red lines indicate increased expression after radiotherapy. Blue lines indicate decreased expression after radiotherapy. Bold line indicates an average expression of 20 patients. Data was analyzed by student t-test. * represents p<0.05. **(E)** RT-PCR and western blot analysis of SIM2l in CvSCC cell lines cells. GAPDH and β-Actin was used as a loading control.

**Supplementary Figure S2**


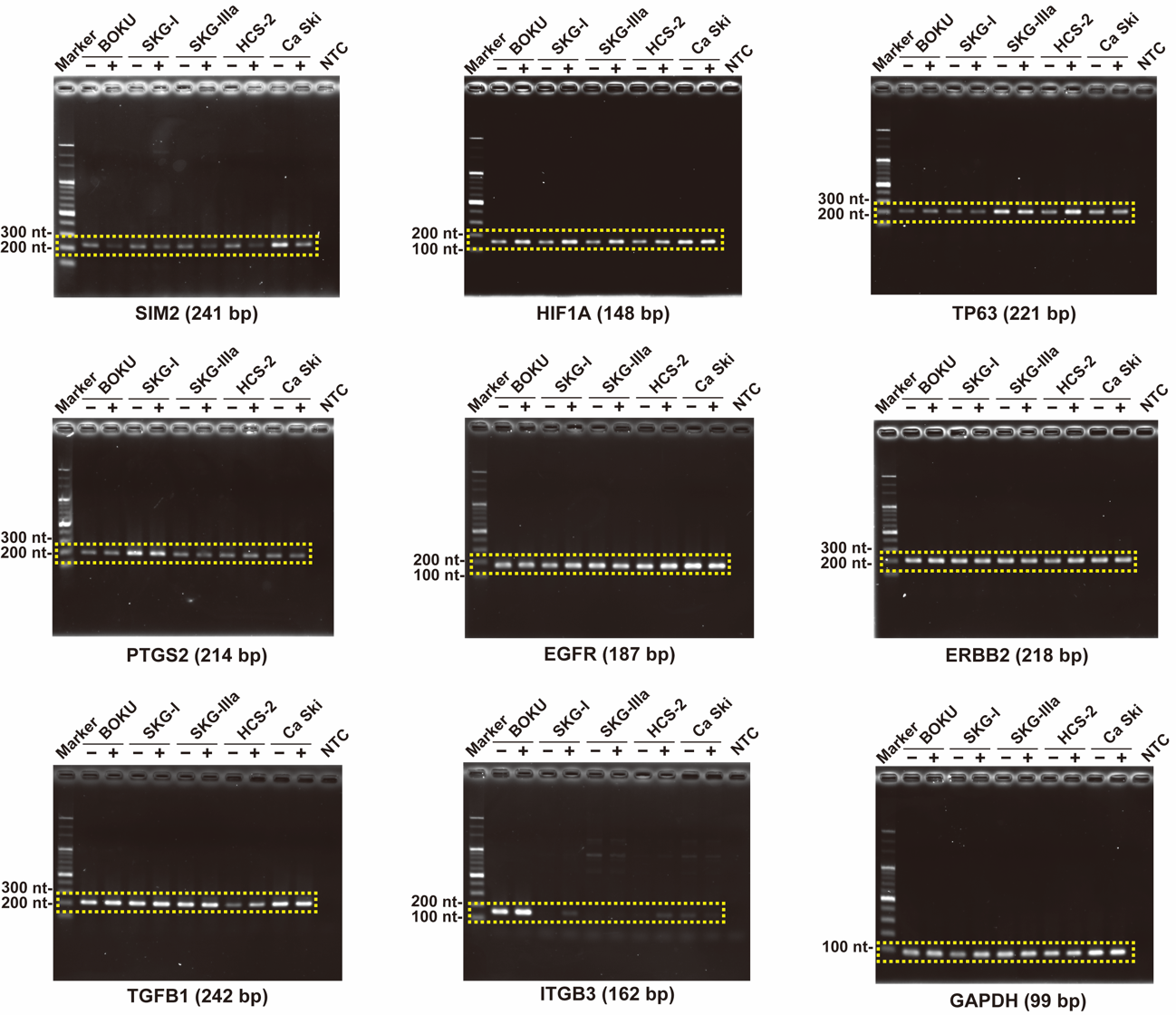


**Supplementary Figure S2. Full length gel images of Figure 2A.**

“-“, “+”, and “NTC” indicates control siRNA, *SIM2* siRNA, and no template control, respectively. Yellow dotted lines show the cropping locations.

**Supplementary Figure S3**


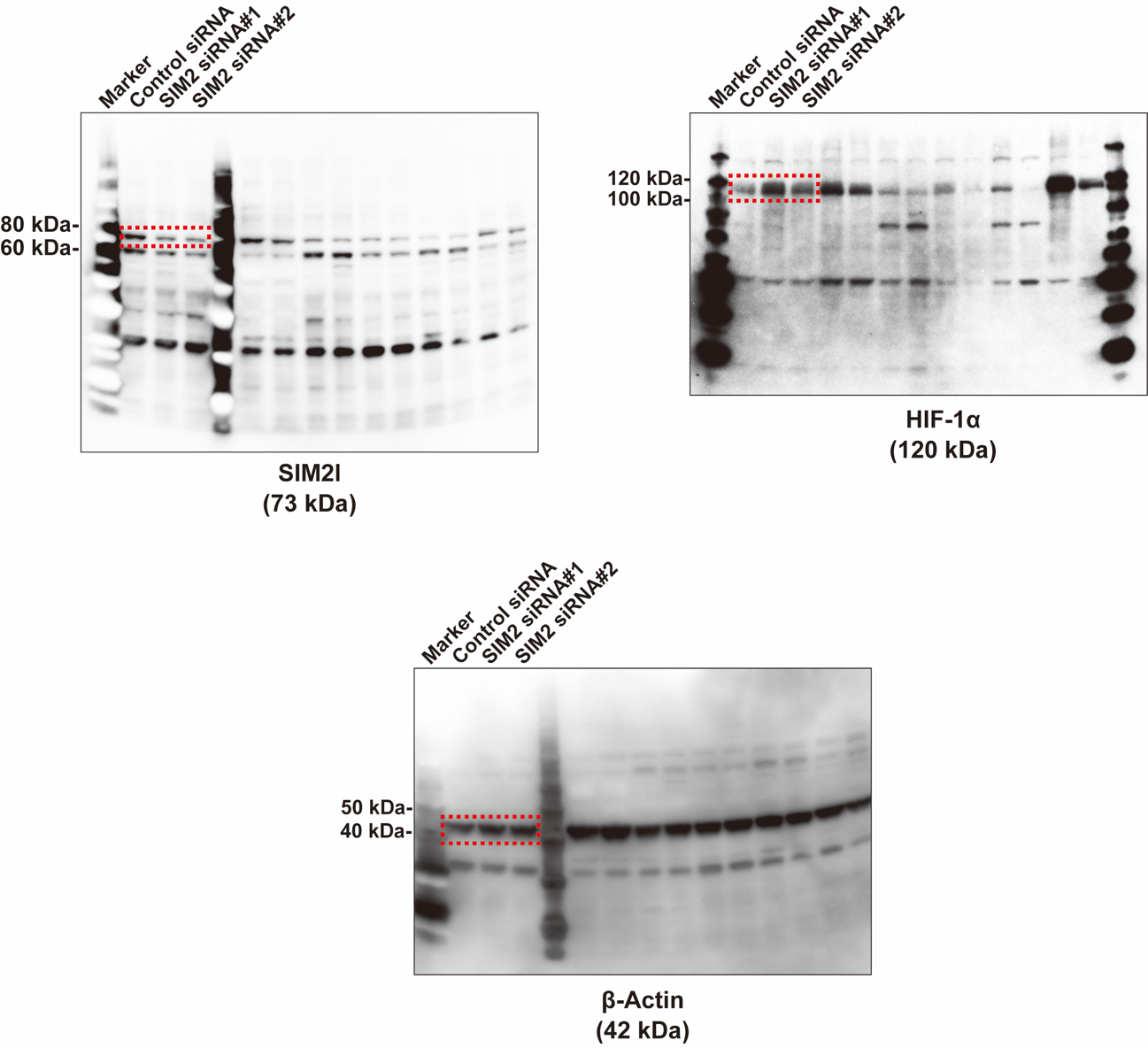


**Supplementary Figure S3. Full length blots of Figure 2C.**

Red dotted lines show the cropping locations.

**Supplementary Figure S4**

**
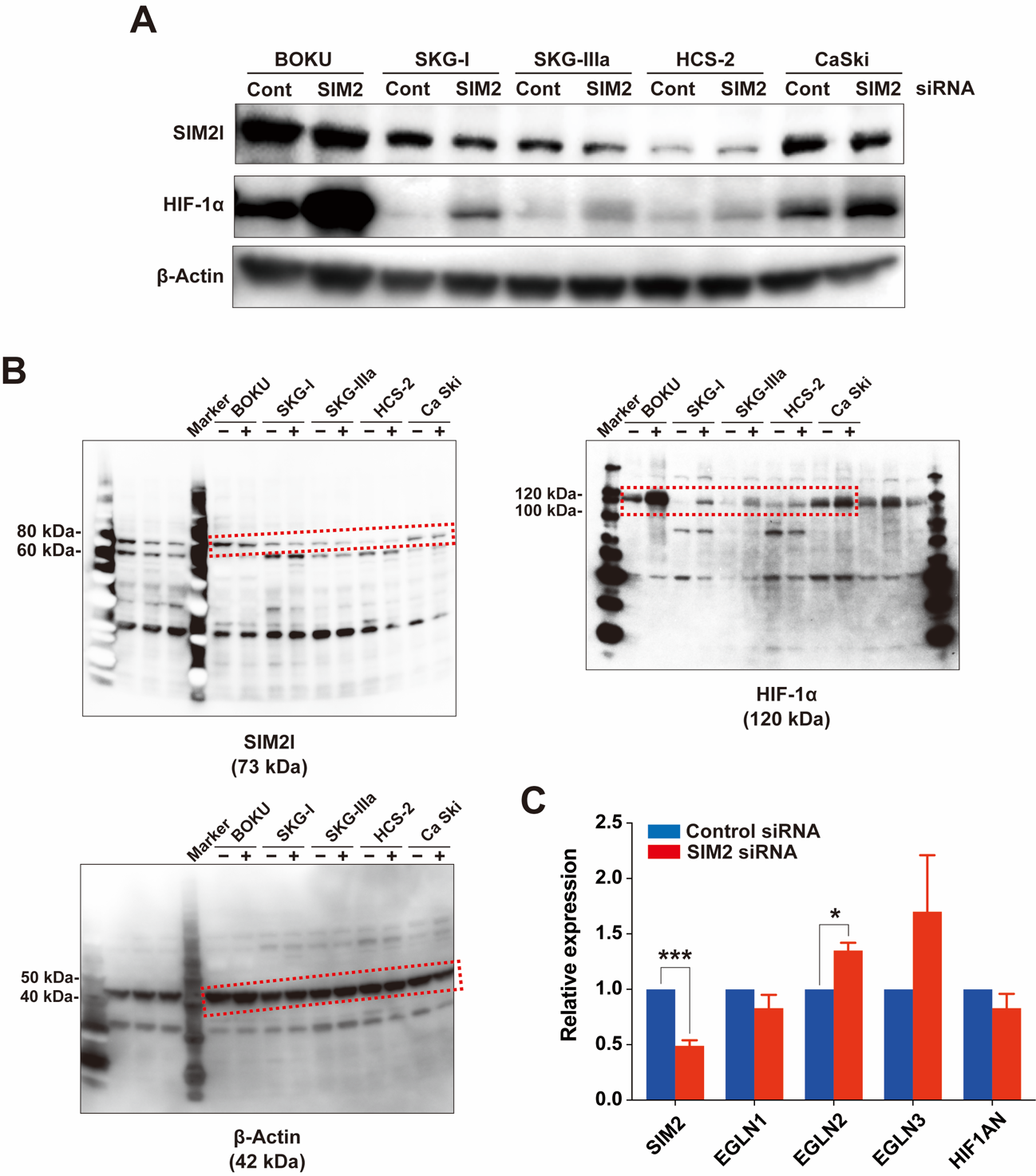
**

**Supplementary Figure S4. SIM2 directly regulates *HIF1A* expression in CvSCC cells.**

**(A)** Western blot analyses of SIM2l and HIF-1α in 5 CvSCC cell lines treated by control siRNA (cont) or *SIM2* siRNA (SIM2). β-Actin was used as a loading control. **(B)** Full blots of (A). “-“ and “+” indicates control siRNA and *SIM2* siRNA, respectively. Red dotte lines show the cropping locations. **(C)** Quantitative RT-PCR analyses of genes encoding proteins to degradate HIF-1α (*EGLN1*, *EGLN2*, *EGLN3*, and *HIF1AN*). All data are obtained from 3 independent experiments and presented as mean±SD. Statistical analyses were performed by student t-test. * and *** represent *p*<0.05 and *p*<0.001, respectively.

**Supplementary Figure S5**

**
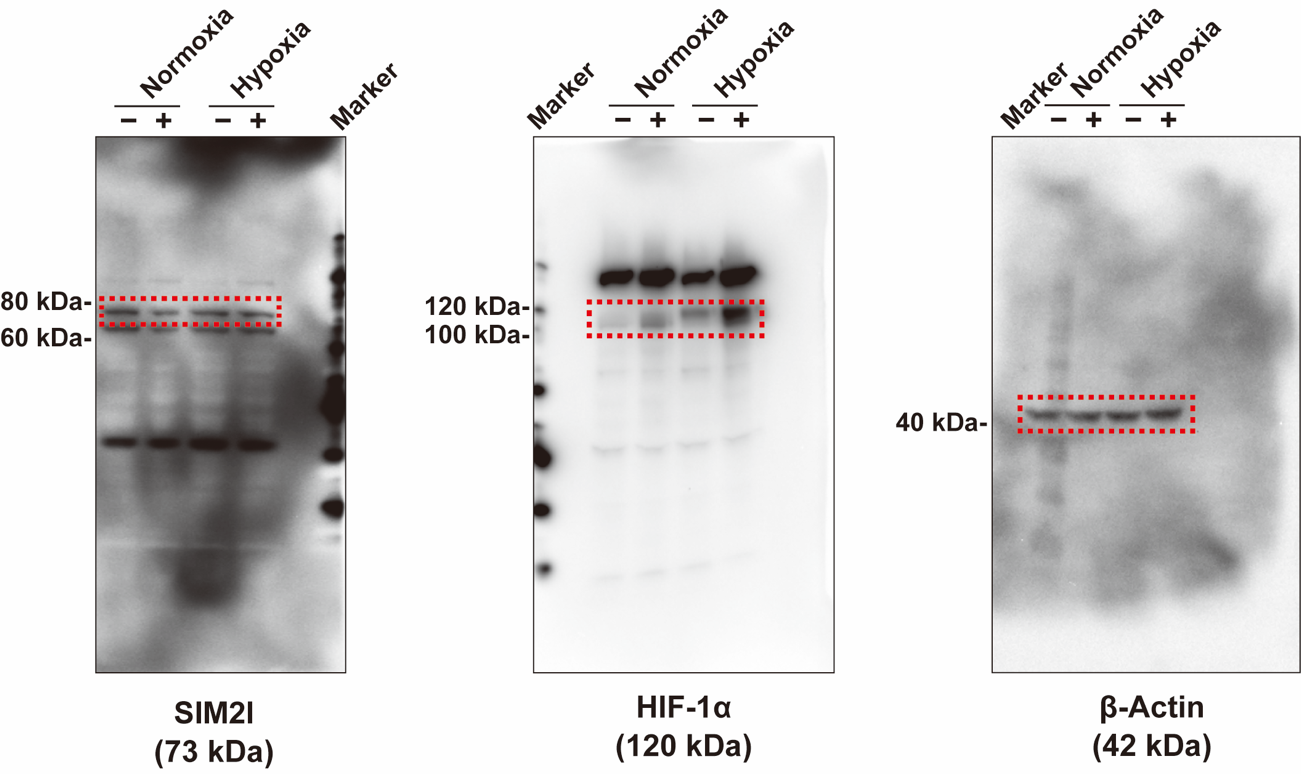
Supplementary Figure S5. Full length blots of Figure 2D.**

“-“ and “+” indicates control siRNA and *SIM2* siRNA, respectively. Red dotted lines show the cropping locations.

**Supplementary Figure S6**


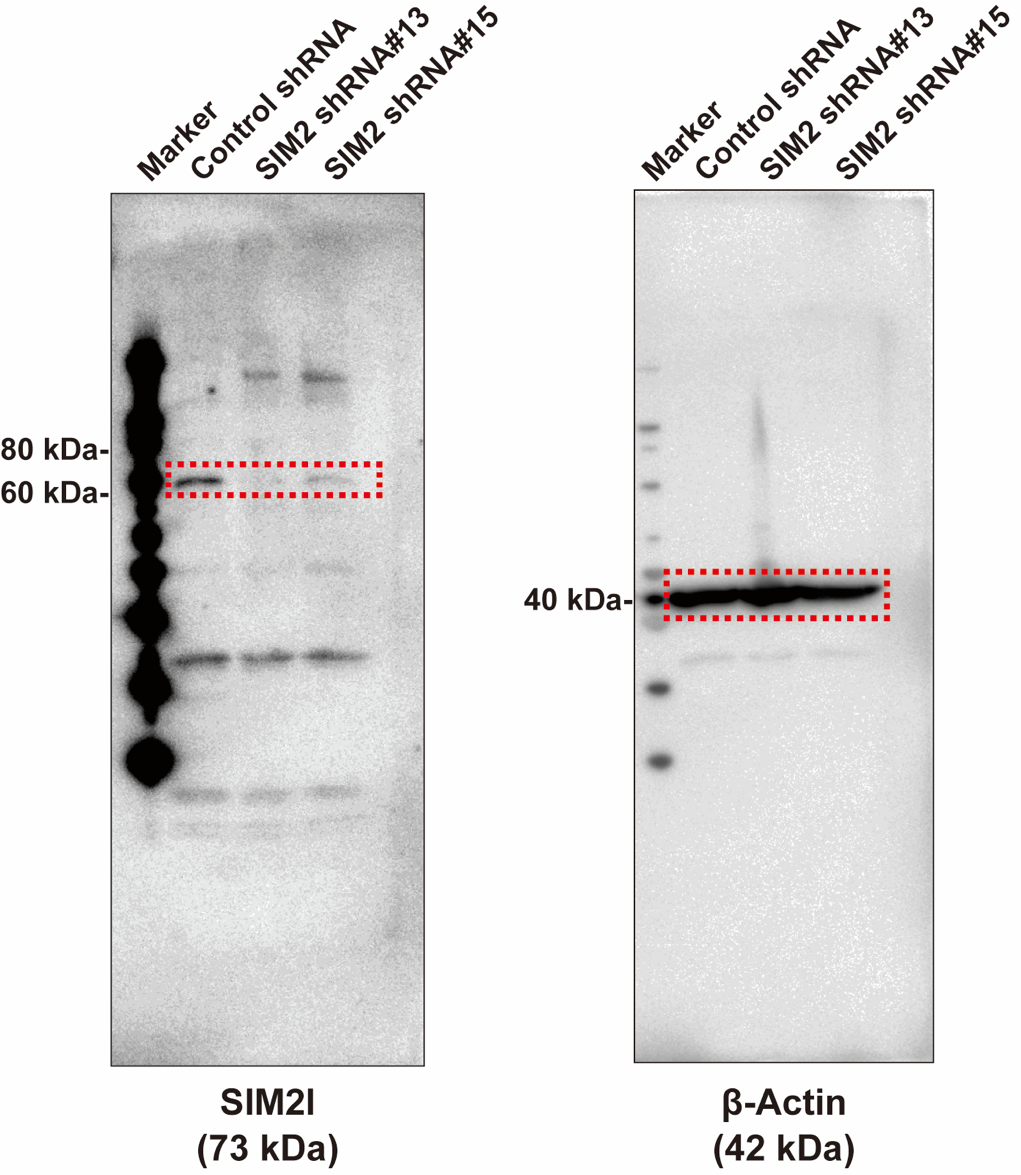


**Supplementary Figure S6. Full length blots of Figure 3A.**

Red dotted lines show the cropping locations.

**Supplementary Figure S7**


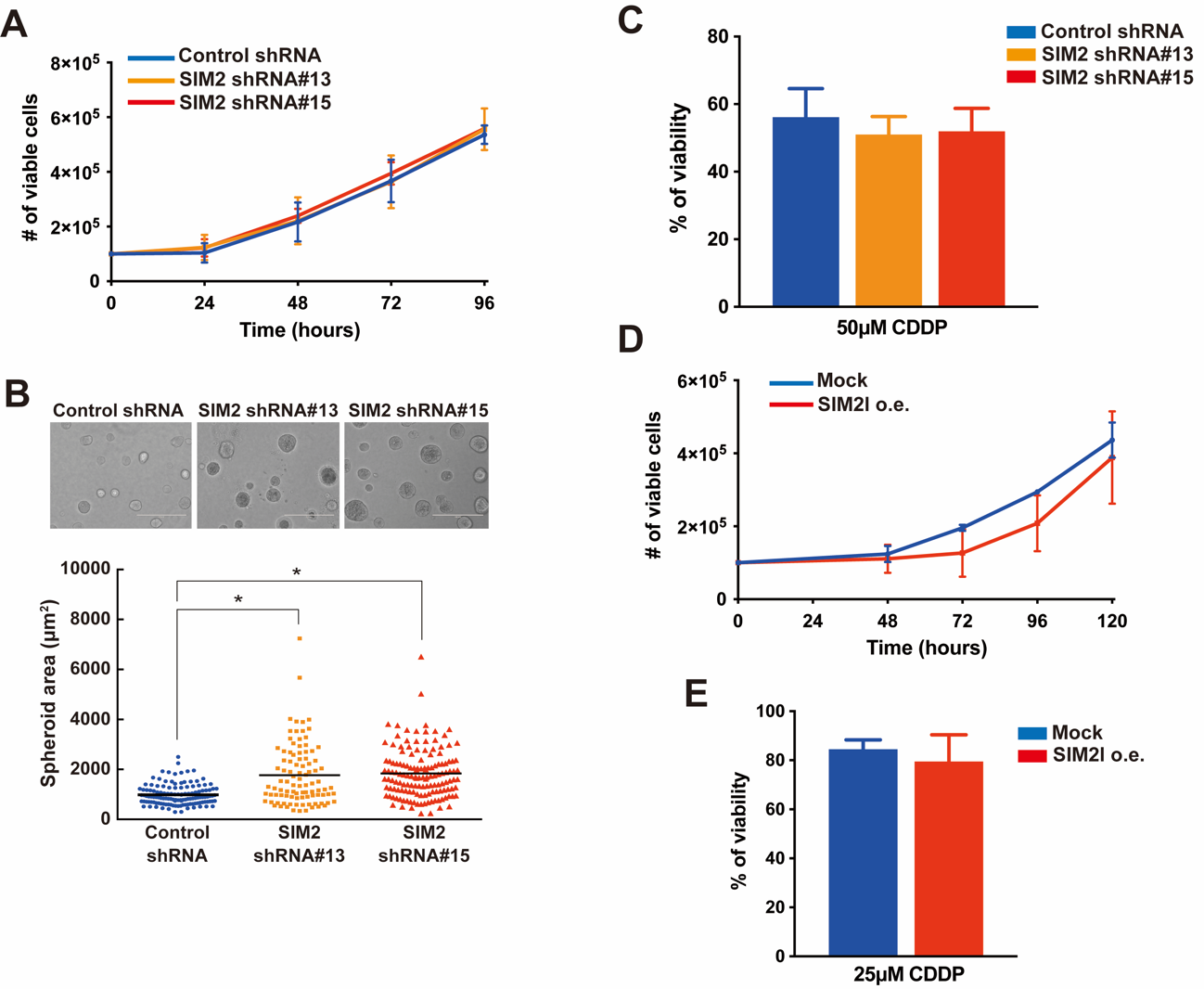


**Supplementary Figure S7. Functinal analysis of SIM2 knockdown and SIM2l overexpression in CvSCC cells**

**(A)** Cell growth curves of a control shRNA-expressing clone mix and two *SIM2* shRNA-expressing clones (#13 and #15). The number of viable cells was monitored every 24 hours. **(B)** Representative bight field images of spheroids in each matrigel (upper) and measurment of their spheroid area (lower). White bars in images are represent 200μm. In a lower panel, each dot represents area of a single spheroid, and black bar is each average. **(C)** Sensitivity to cisplatin (CDDP) under a 3D culture of SIM2-knockdown cells. Cells were cultured for 7 days in the absence/presence of 50μM CDDP. Each result is normalized by the viability of untreated control. **(D)** Growth curves of mock vector-transfected and SIM2l-overexpressing SKG-Ι cells (Mock and SIM2l o.e.). **(E)** Sensitivities of SIM2-overexpressing cells to CDDP under a 3D culture. Each cell viability at 9 days is normalized by mock-transfectants.

Out of (**C**), all data were obtained from 3 independent experiments and presented as mean±SD.

**Supplementary Figure S8**


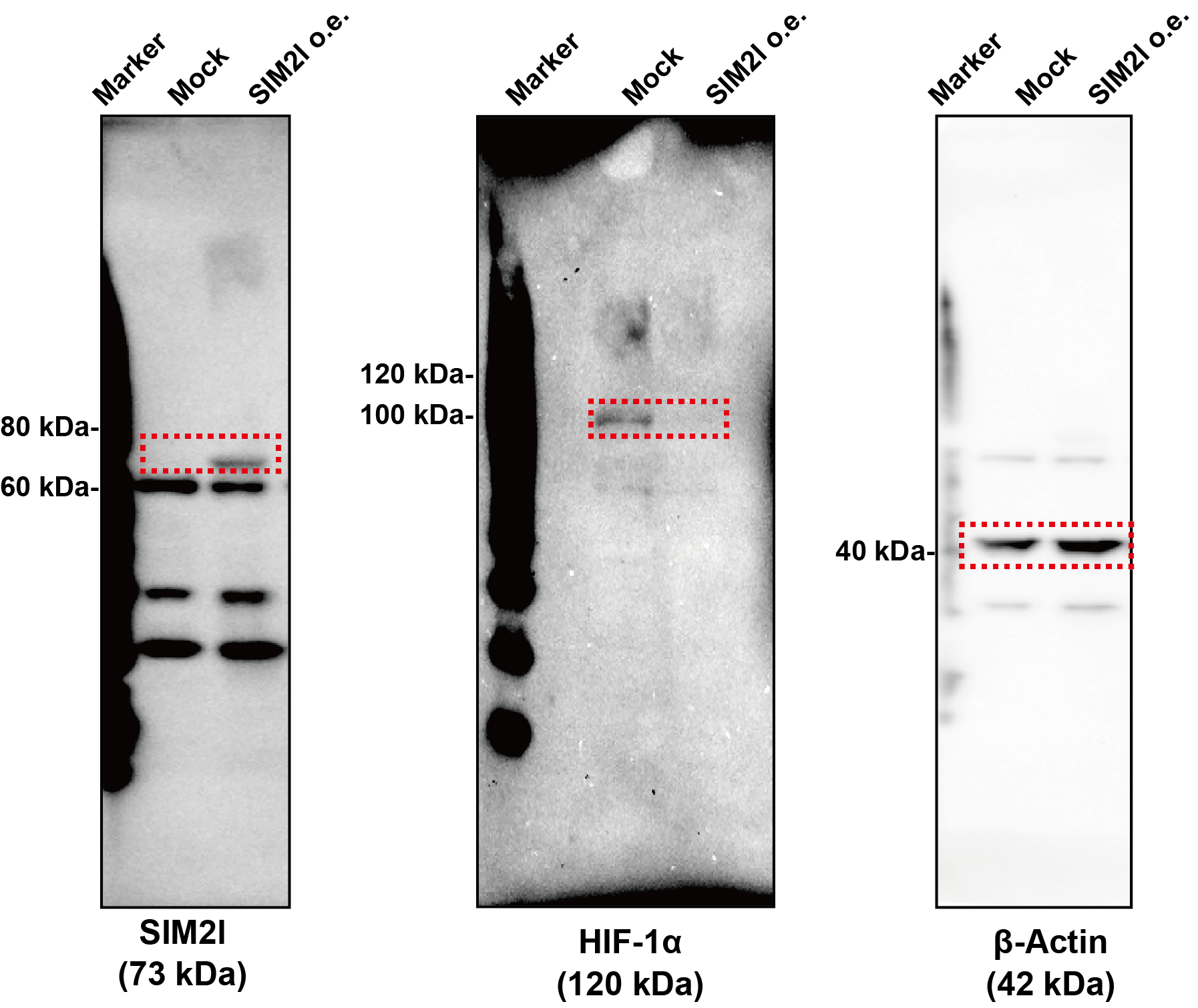


**Supplementary Figure S8. Full length blots of Figure 5A.**

Red dotted lines show the cropping locations.

**Supplementary Figure S9**


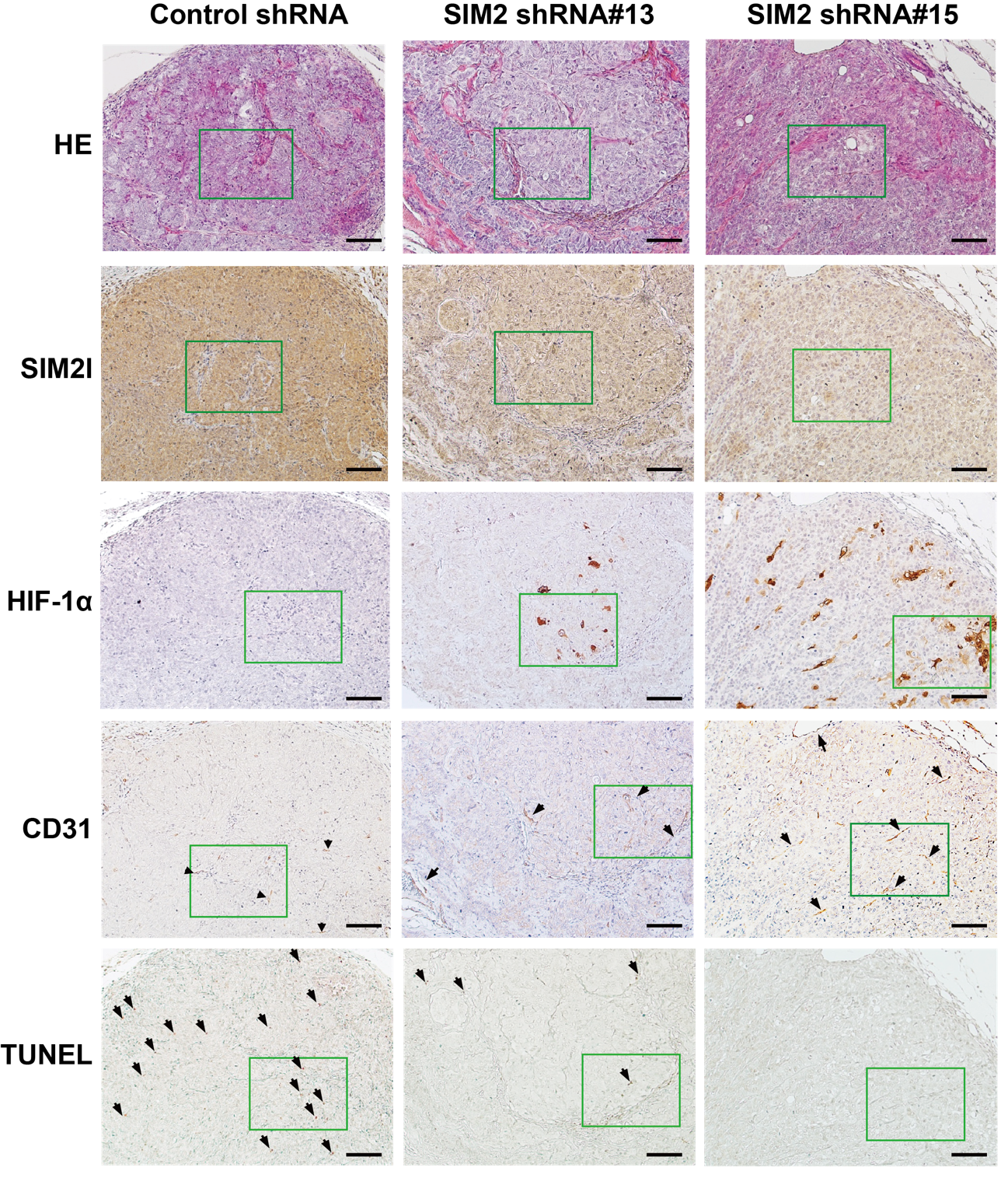


**Supplementary Figure S9. Full images of Figure 4C.**

Green dotted lines show the cropping locations.

**Supplementary Figure S10**


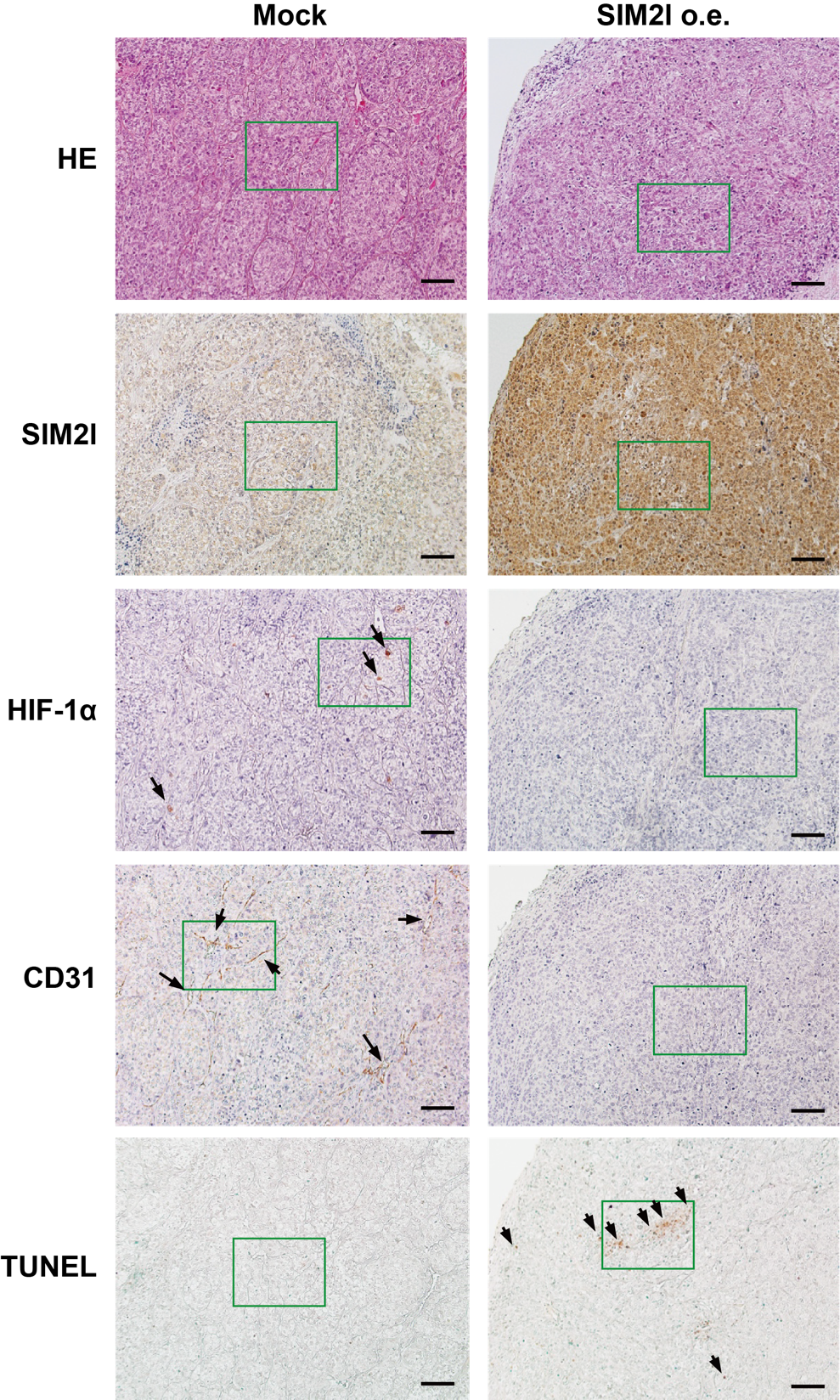


**Supplementary Figure S10. Full images of Figure 5F.**

Green dotted lines show the cropping locations.

**Supplementary Table S1**

**Supplementary Table S1.** Comparison of xenograft tumor size between control shRNA and SIM2 shRNA-expressing cell lines.

^a^ Statistical significance between control shRNA and SIM2-expressing cell lines.

**Supplementary Table S2**

**Supplementary Table S2.** Comparison of xenograft tumor size between mock and SIM2l-overexpressing cell line.

^a^ Statistical significance between mock and SIM2l-overexpressing cell line.

**Supplementary Table S3**

**Supplementary Table S3.** List of primer sequences


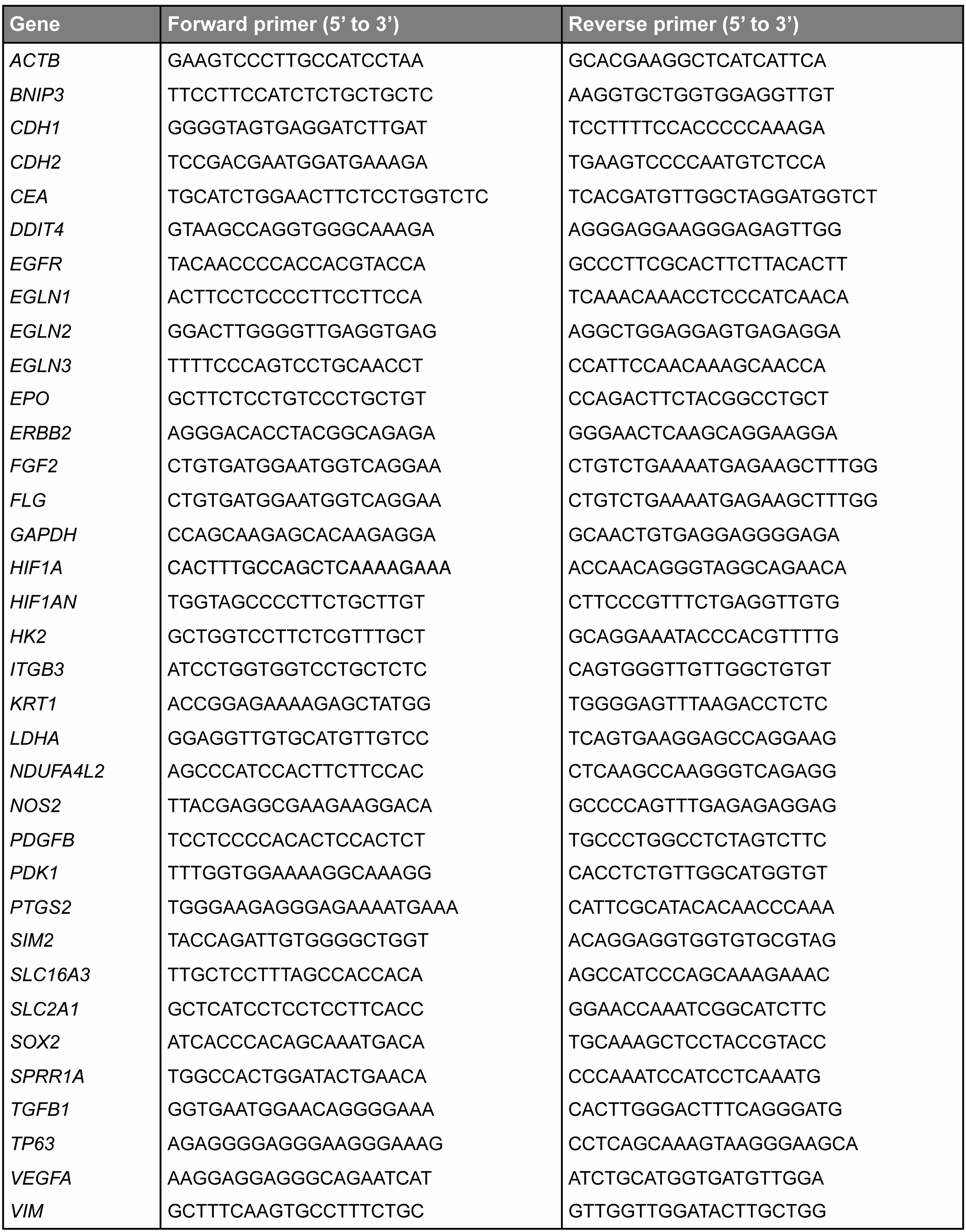

Supplement: Supplementary file 1 — Supplementary Information [file 41598_2017_15261_MOESM1_ESM.doc]
